# Modification of plasmonic properties in several transition metal-doped graphene studied by the first principles method†

**DOI:** 10.1039/d2ra06446d

**Published:** 2023-01-05

**Authors:** Diyan Unmu Dzujah, Abdul-Muizz Pradipto, Rahmat Hidayat, Kohji Nakamura

**Affiliations:** a Physics of Magnetism and Photonics Research Division, Physics Study Program, Faculty of Mathematics and Natural Sciences, Bandung Institute of Technology Jl. Ganesha 10 Bandung Indonesia rahmat@fi.itb.ac.id; b Department of Physics Engineering, Faculty of Engineering, Graduate School of Engineering, Mie University Tsu Mie 514-8507 Japan

## Abstract

Graphene doped with different transition metal (TM) atoms, namely, Co, Ni, Cu, Zn, and Au, have been investigated through first-principles calculations. The TM atom forms a substitutional defect, replacing one carbon atom in the graphene basal plane, which considerably can be obtained through wet or dry chemical processes as reported elsewhere. The calculation results showed that TM atom substitution leads to the opening of a band gap and the emergence of mid-gap states with the Fermi energy in the middle of it. The effects on optical properties were seen from the calculated optical absorption and Electron Energy Loss Spectroscopy (EELS) spectra. Two EELS bands are seen in the far UV region corresponding to the π and (π + σ) plasmons but the influence of the substituted TM effects on the plasmon frequency is small. On the other hand, as the Fermi energy level appears in the middle of the mid-gap state band while the real part of its dielectric permittivity at low photon energy is negative, these TM-doped graphene have a metal-like characteristic. Hence, plasmon wave excitation can be expected at the THz region which is dependent on the dopant TM atom. The plasmon excitation in these TM-doped graphene is thus principally similar to the plasmonic excitation in pure graphene by electric or magnetic fields, where the Fermi energy level is shifted from the graphene Dirac point leading to the possibility of an intraband transition.

## Abstract 2

Doping graphene with a single transition metal atom results in bandgap opening and partial filling of the mid-gap states, leading to the possibility of plasmon excitation with plasmon frequency depending on the TM dopant.

## Graphical abstract

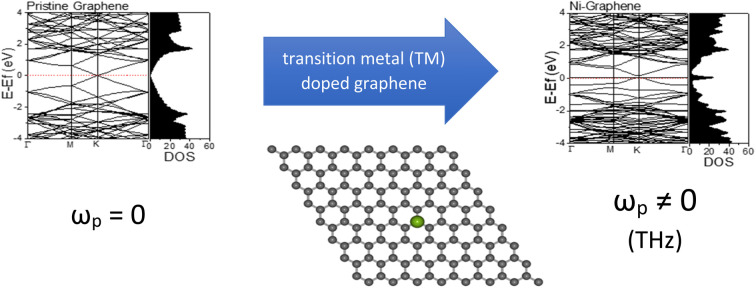

## 1. Introduction

Graphene is a two-dimensional (2D) carbon allotrope possessing an sp^2^-hybridized honeycomb structure that exhibits excellent electrical and optical properties owing to its electronic structure with a Dirac cone shape. This Dirac cone is formed from a cross-section point of the valence and conduction bands, resembling the stacking of two cones in opposite orientations, creating a zero bandgap and zero density of states (DOS) at the cross-section point. Because the Fermi energy is located at the cross-section point, *i.e.*, the Dirac point, graphene is a semi-metal with zero-bandgap semiconductor properties. As already reported elsewhere, these unique properties have led to diverse applications of graphene.^1–7^ Initially, benefitting from the electric field effect in 2D materials, graphene was used in transistor-like devices that attracted much attention.^1,2^ Subsequently, other physical properties of graphene have also been reported and used for different potential applications. Among others, owing to its high electron mobility and catalytic properties, graphene has been applied as an electrode material in supercapacitors, batteries, solar cells, and electrochemical-based sensors.^2,8–10^

The plasmonic properties of graphene have also been intensively studied because of its strong light–matter interactions.^11–14^ Graphene has advantages as a plasmonic material because of its low losses and large electromagnetic field confinement.^15,16^ However, in pristine (undoped) graphene, being a perfect semi-metal, plasmonic wave cannot be generated directly due to zero charge carriers at its Dirac point. It should be noted that the real part of the dielectric permittivity of pristine graphene is negative in the mid-infrared (mid-IR) to THz frequency region. In general, the real part of the dielectric permittivity must be negative for metal-based plasmonic materials. Several methods have been proposed and applied to attain plasmonic excitation and resonance tuning in graphene, such as by using multilayer structuring, in order to match the momentum between incident light and the electrons in graphene, and other different approaches through electric and magnetic fields, mechanical strain, and impurities/defects insertion, in order to shift the Fermi energy from the Dirac point.^17–26^ Most studies on the utilization of surface plasmon polariton (SPP) waves in graphene are performed in the mid-IR to terahertz (THz) frequency range. THz sensors and detectors are important in THz spectroscopy that have found a wide range of applications in biomedical imaging, biochemical sensing, environmental monitoring, *etc.*^27,28^ Using THz spectroscopy, one could investigate low-energy molecular vibrations, crystal-lattice or phonon vibrations and intermolecular vibrations of macromolecular structure hydrogen-bonded network structure.^29^ Zheng *et al.* have investigated both experimentally and computationally the vibrational spectra of benzene-1,2-diol, in which they concluded that the spectrum peaks in the mid-infrared range can be associated with intramolecular vibration whereas the peaks in THz range can be associated with intermolecular vibrations.^30^

In addition to the plasmon at low energy in mid-IR to THz frequency range as mentioned above, there is another plasmon with much higher photon energy, in the far UV region, that also becomes a subject of interest. Experimentally, the presence of plasmons in several materials has been extensively investigated using electron energy loss spectroscopy (EELS). Kuzuo *et al.* have performed EELS on carbon nanotubes, reporting a π plasmon at either 5.2 or 6.4 eV and a (π + σ) plasmon at 22–24.5 eV.^31^ Reed *et al.* then investigated isolated single-walled carbon nanotubes and reported spectral bands associated with π plasmon at approximately 4.5–5.5 eV and (π + σ) plasmon at 13–27 eV.^32^ By the same principle, EELS has also been employed to investigate the plasmon characteristics in graphene, in which two broad plasmonic bands were detected at around 5 eV and 10–35 eV for the π and (π + σ) plasmons, respectively.^33–35^ The π plasmon originates from the π–π* transition, while the (π + σ) plasmon originates from higher photon energy transitions, such as σ–σ*, π–σ*, and σ–π* transitions. Besides experimental investigations, numerous efforts have been also devoted to predicting the plasmonic properties of pristine graphene through first-principles calculations.^26–28,36^

Recently, Zhang *et al.* demonstrated the formation of Ni single-atom bonding with a defect vacancy in a 2D graphene sheet, in which the defect was created through an impregnation method with acid leaching.^37^ Jiang *et al.* reported direct evidence of the coordination bonding formation of Ni single atoms with graphene vacancy defects using scanning transmission electron microscopy (STEM).^38^ Besides the synthesis *via* a wet chemical process, another approach *via* a dry chemical process has also been reported. Dyck *et al.* then demonstrated the formation of Ni-doped graphene by using an electron beam, where the Ni adatom can be selectively and precisely inserted into a graphene vacancy defect.^39^ Single-atom B or N-doped graphene achieved by low-energy ion implantation has been reported by Hage *et al.*, in which a dampening or enhancement of the characteristic interband π plasmon was observed.^40^ The defect resembles a substitutional defect where the metal atom replaces a carbon atom in the basal plane. Motivated by those reports, we have performed first-principles calculations based on density functional theory (DFT) methods to investigate the effects of the substitution of different TM atoms (Co, Ni, Cu, Zn, and Au) on the electronic structure, optical properties, and plasmon characteristics of 2D graphene sheets (hereafter called as TM-doped graphene). The results demonstrate that TM doping leads to bandgap opening and Fermi energy shift in their electronic structures as well as the possibility and modification of THz plasmon excitation.

## 2. Methodology

**Fig. 1:**
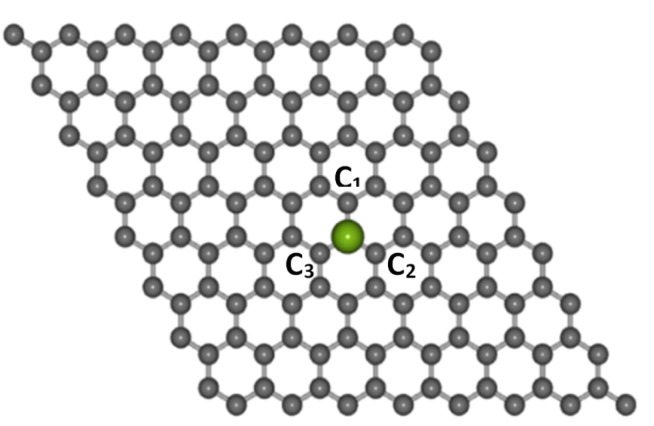
The structures of Co-doped graphene around the TM dopant atom (purple color ball) after structure relaxation calculations.

Electronic structure computations were conducted using the DFT method,^41,42^ which was implemented in the Quantum Espresso package.^43^ The generalized gradient approximation (GGA) along with the Perdew–Burke–Ernzerhof (PBE) exchange-correlation functional and the projected augmented wave (PAW) pseudopotentials were used.^44,45^ The cut-off energy was set to 525 eV. The transition metal atoms considered as dopants in this study were Co, Ni, Cu, Zn, and Au. Each dopant was substituted at the center of a graphene supercell consisting of 8 × 8 unit cells (127 carbon atoms and 1 dopant atom), as shown in Fig. 1, with a 10 Å vacuum layer separator in the *z*-direction to eliminate the interaction between layers. A *k*-point mesh of 9 × 9 × 1 was used for the calculations.

First, each TM-doped graphene structure was optimized until the change in energy was lower than 10^−8^ Ry. The lattice constant for graphene was 1.42 Å, as reported in the literature.^46^ The relaxation of atomic position in the unit cell was performed by using the Broyden–Fletcher–Goldfarb–Shanno (BFGS) algorithm. Relaxation was allowed in the *x* and *y* directions. A slightly larger *k*-point mesh of 11 × 11 × 1 was applied for the calculation of DOS and PDOS, and 20 points along the *Γ*–*M*–*K*–*Γ* high symmetry *k*-path were considered to obtain the band structures. Optical properties were calculated using the optimized norm-conserving Vanderbilt (ONCV) pseudopotential with *Γ*-centered calculation.^47–49^

From those calculated electronic structures, the optical properties were then calculated in a sequential step initiated from the calculations of the dielectric function, absorption spectra, and finally the EELS spectrum calculation.

## 3. Results and discussions

### 3.1. Structure optimization and electronic structure

**Fig. 2:**
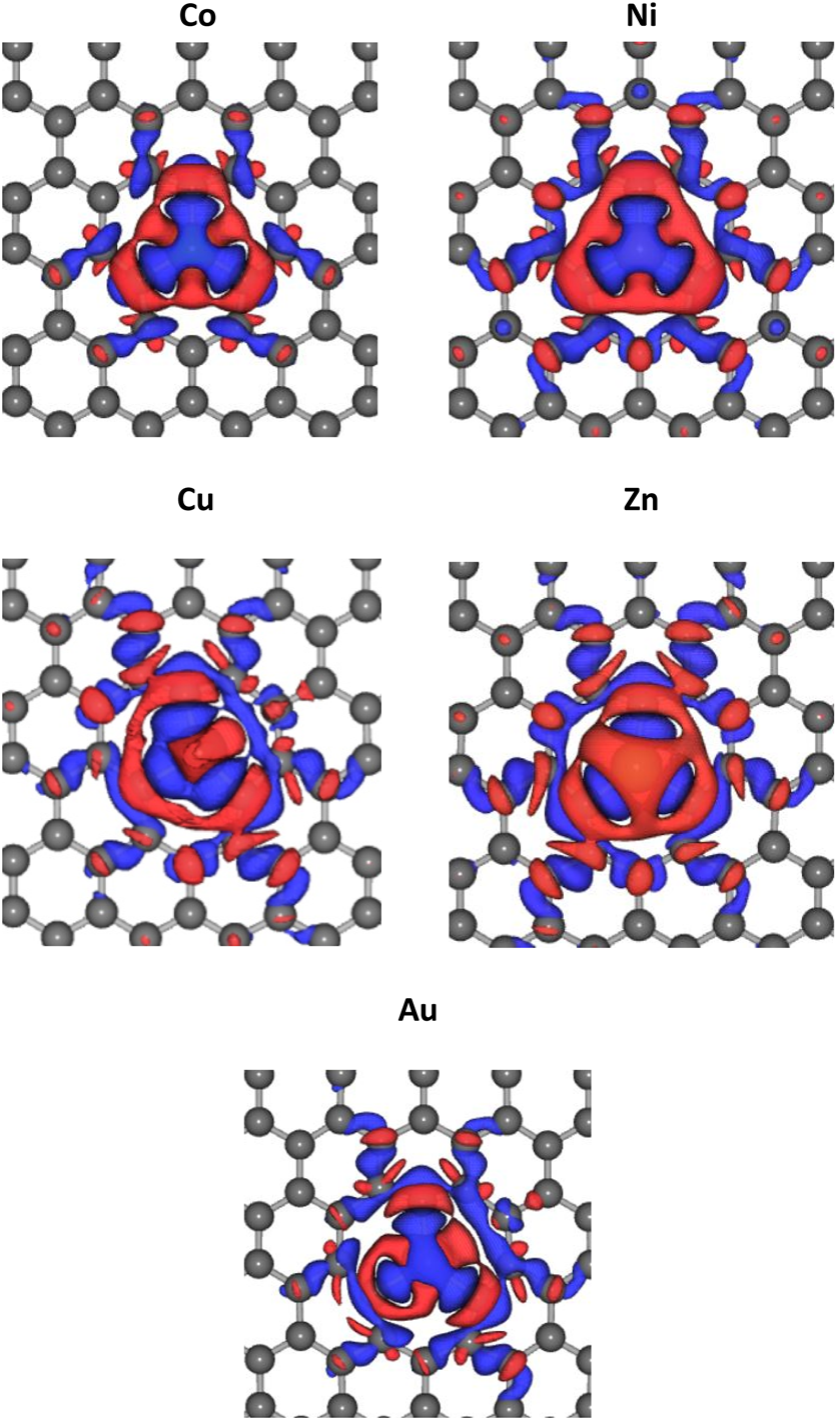
Change of electric charge density upon TM substitution into the graphene. Blue and red iso-surfaces (0.001 e Å^−3^) indicate electron depletion and electron accumulation, respectively.

The optimized lattice structures were obtained after the structure relaxation calculations converged, that is, when the minimum energy for each calculation has been reached. Our structure optimizations confirm that a single TM atom can form bondings with the three nearest C atoms, as depicted in Fig. 1. The distances between C and TM (*d*_C–TM_) after structure optimization for these TM-doped graphene are presented in Table 1. A larger C–TM bond length is seen for TM with larger covalent radii (Ni = 125 pm, Co = 125 pm, Zn = 137 pm, and Au = 144 pm). For comparison, the C–C bond distance is 1.45 Å while the C covalent radius is 77 pm. TM atoms with higher covalent radii cause more local lattice deformation around the TM atom. Changes in the electric charge distributions due to TM substitution were investigated by Bader analysis using the charge density difference definition as follows:

1 Δ*ρ*_TM_ = *ρ*_TM-doped graphene_ − *ρ*_graphene_ − *ρ*_TM_

where *ρ*_TM-doped graphene_, *ρ*_graphene_, and *ρ*_TM_ are the charge densities of TM-doped graphene, pristine graphene with vacancies, and TM atoms, respectively.^18,57,58^ As shown in Fig. 2, the electron density of the TM atom decreased after TM-doping (blue iso-surface in Fig. 2). Moreover, the electron density around the three nearest C atoms to the TM atom increased (indicated as the red iso-surface, Fig. 2). These behaviors confirmed bond formation between the TM and its nearest C atoms.

**Table 1:** Structural parameters from optimization results of the TM-doped graphene with the neighboring carbon atoms

Structure	*d*_C1–TM_ (Å)	*d*_C2–TM_ (Å)	*d*_C3–TM_ (Å)
Co-doped graphene	1.67	1.67	1.67
Ni-doped graphene	1.70	1.70	1.70
Cu-doped graphene	1.67	1.67	1.83
Zn-doped graphene	1.71	1.71	1.78
Au-doped graphene	1.84	1.84	1.85

**Fig. 3:**
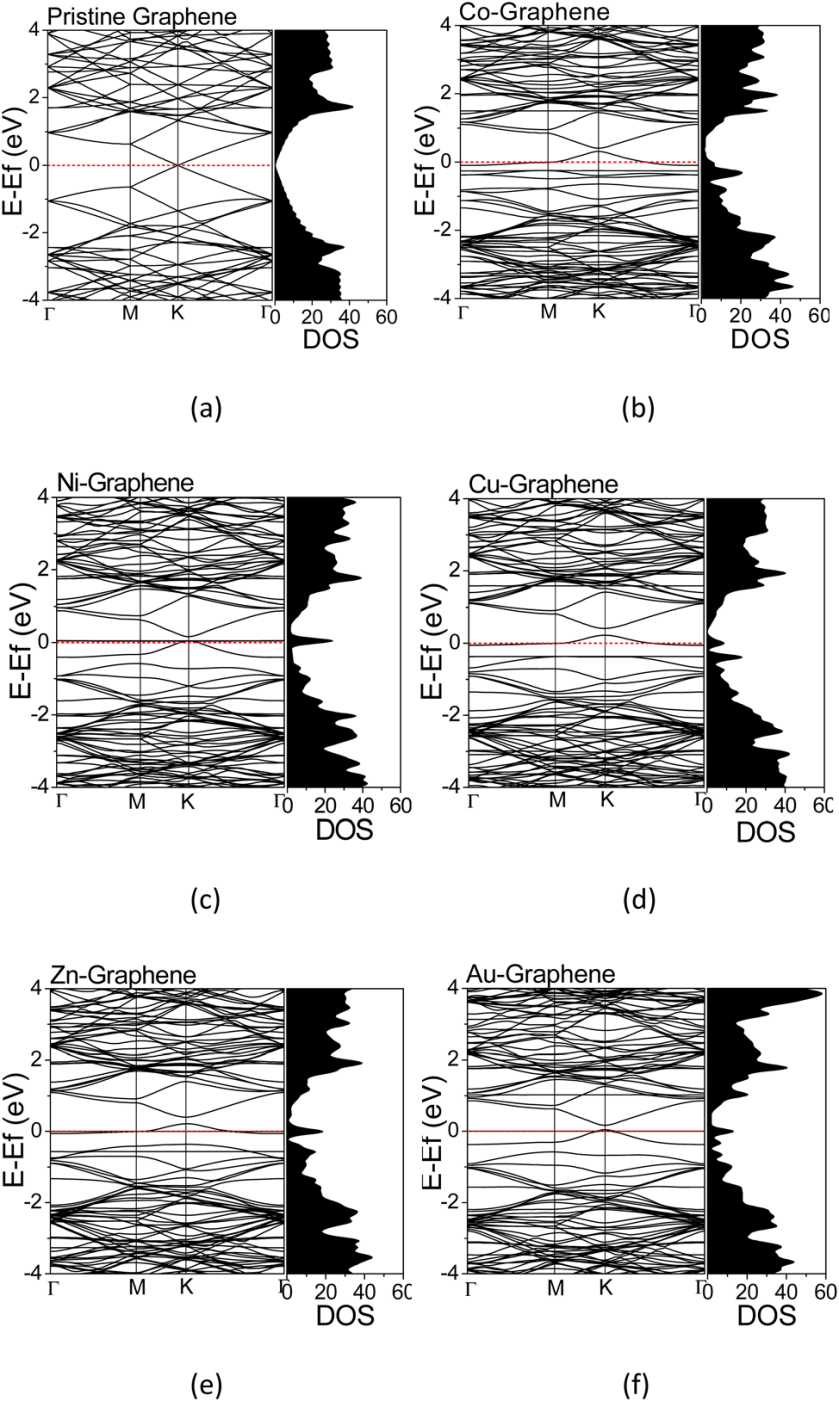
Electronics structures and DOS of (a) pristine graphene; (b) Co-, (c) Ni-, (d) Cu-, (e) Zn-, and (f) Au-doped graphene.

**Fig. 4:**
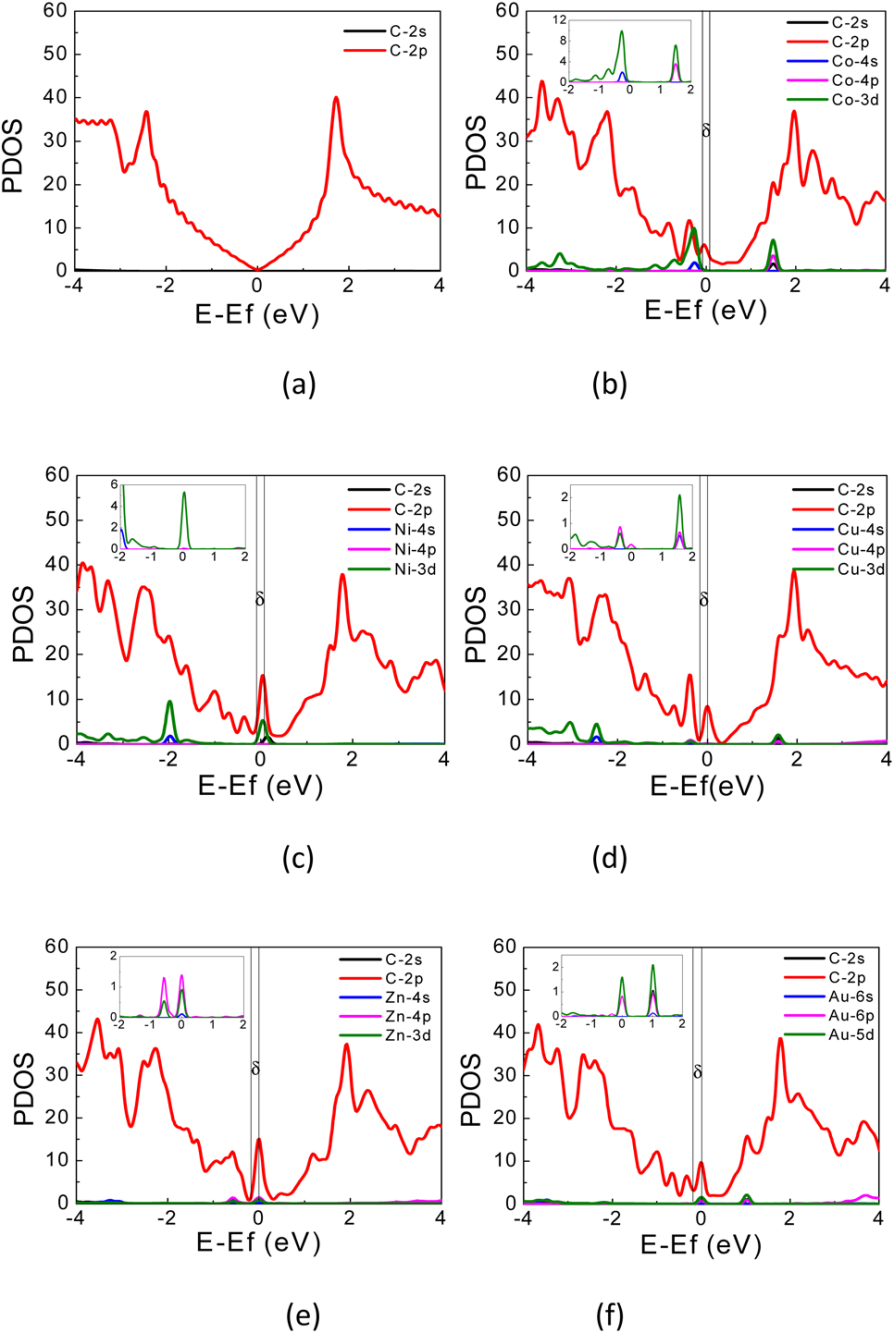
PDOS of (a) pristine graphene; (b) Co-, (c) Ni-, (d) Cu-, (e) Zn-, and (f) Au-doped graphene. Fermi energy is located at 0 eV.

The electronic structures were calculated along the high-symmetry *Γ*–*M*–*K*–*Γ* path on the Irreducible Brillouin Zone (IBZ). The calculated electronic structures provided information regarding the electron states at a particular wavenumber (*k*) and energy in the TM-doped graphene. The calculated electronic structures and the corresponding DOS for graphene and TM-doped graphene are shown in Fig. 3. The energy scale has been shifted such that the Fermi level is located at 0 eV. For pristine graphene, *i.e.*, without dopant (Fig. 4(a)), the calculated electronic structure exhibited a Dirac cone shape at the high-symmetry point of *K*, in which the Fermi energy (*E*_F_) level is located at the Dirac point. This result was in agreement with that reported in the literature, demonstrating the calculations' validity herein.^22,43,50^ Significant changes in the electronic structures of TM-doped graphene can be seen in Fig. 3(b–e), along with the corresponding DOS profiles in comparison to those of pristine graphene. There is a bandgap opening at the Dirac point, and the Fermi energy is also shifted downward from the Dirac point into the valence band, where the magnitude of the shift varies for different TM dopants. The band gaps are about 0.084, 0.109, 0.193, 0.192, and 0.116 eV for Co, Ni, Cu, Zn, and Au doped graphene, respectively. For the Co-doped graphene, the Fermi energy level remarkably shifts downward to the valence band. However, in the case of Ni and Au dopants, the Fermi energy level just shifts slightly downward to the valence band. For all TM-doped graphene, a narrow DOS band δ appears at the Fermi energy level, where it does not appear in pristine graphene. This narrow band can be associated with mid-gap states formed mutually due to the formation of C–TM bonds and the local structural alteration of C–C bonds surrounding the TM atoms.

The PDOS curves demonstrate the contribution of specific orbitals to the electronic band. The PDOS presented in Fig. 4 confirmed that the mid-gap states band δ is constructed predominantly by the 2p orbitals of the carbon atoms and TM orbitals. However, as seen in Fig. 4(b), for Co-doped graphene, the Co-3d orbital also contributes to the formation of this mid-gap band at the same energy level as the C-2p orbital, indicating extensive hybridization that might be resulted from coordination bonding formation. In this case, the π-electrons from the surrounding carbon atoms are shared with the TM atom. This may explain the shifting of the Fermi energy level to a lower energy level leading to the topmost valence band level at the mid-gap states. In the case of Zn, the mid-gap states band δ around the Fermi energy level also appears, but it is contributed dominantly only by the 2p orbitals of the carbon atoms. The contribution from Zn orbitals is not seen, which may indicate that the C–Zn bond is formed without involving its 3d orbitals, plausibly because the 4s and 3d orbitals of Zn are already filled. This may affect the charge density difference distribution in the Zn-doped graphene, exhibiting a different profile compared to that found in Co-doped graphene.

For Au-doped graphene, the contribution of Au orbitals in the mid-gap state band δ is very little, similar to the Zn dopant, because the formed bonds do not involve the Au 3d orbitals but likely other orbitals with much lower energies than the Fermi energy. In this case, the mid-gap band is then formed merely due to the structural/bond alteration of the graphene units surrounding the TM atoms. As the mid-gap states also involve valence electrons from carbon atoms of this graphene, the band structure now forms a metal-like band structure, where the states below the Fermi energy are filled but states above it are empty. Despite the number of free electrons will be small, we may still expect metal-like properties and hence plasmonic characteristics, where the plasmonic frequency will be determined by this free electron density.

It is worthwhile to note that similar systems consisting of a single transition metal atom embedded in nano-molecules or two-dimensional architectures have been also reported, which are mainly intended for developing efficient catalytic materials.^51–54^ In systems with low electron delocalization, the valence electrons from both TM and the ligand or surrounding atoms may form a bonding through several possible hybridization schemes leading to a typical trigonal bipyramidal or octahedral local coordination. In such cases, analysis of the ligand field effect should be carefully undertaken, which may determine the orbital level splitting as well as the spin multiplicity.^55^ For the cases discussed here, the PDOS for these TM-doped graphene and the electric charge difference Δ*ρ*_TM_ indicate that the bonding is also formed from a hybridization scheme which involves involving the 3d, 4s, and 4p orbitals of the TM atom and the 2p orbitals from surrounding C atoms. However, in contrast to the previous studies,^51–54^ electrons in these TM-doped graphene are much more delocalized, as implied by the local charge in each atom. Tables S1–S6 of ESI† indicates that the charges in each carbon atom and TM atom are smaller than their valence numbers, suggesting that a portion of electrons are largely delocalized around the TM dopant. Such electron delocalization leads to unfavorable splitting between up- and down-spin densities. Additionally, the PDOS shows low densities of states of the transition metal 3d orbitals as the spin carrier, which does not satisfy the Stoner criterion leading to zero total magnetization.^56^ Tables S1–S6 of the ESI† also tabulates the magnetic moments in each atom and the total magnetizations, which are very small within our computational accuracy. The spin multiplicity may thus be neglected as a first approximation, in contrast to the previous studies on the low electron delocalization systems.^51–54^ For the TM-doped graphene considered in this work, it seems that spin multiplicity has a minor role in the electronic structure and the subsequence optical properties (see ESI Point no. 2†).

### 3.2. Optical and plasmonic properties

**Fig. 5:**
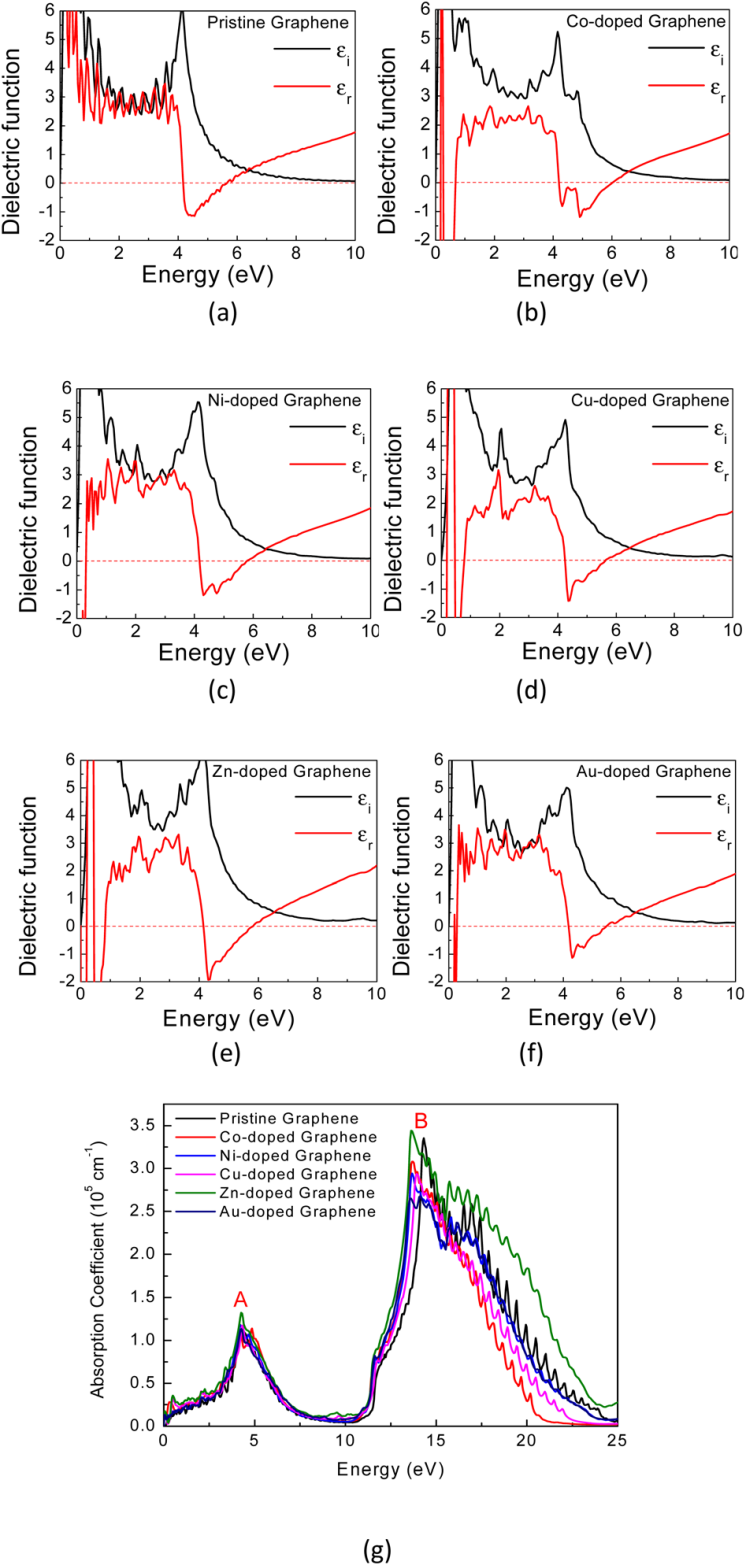
The calculated dielectric permittivity and absorption spectra of (a) pristine graphene, (b) Co-, (c) Ni-, (d) Cu-, (e) Zn-, and (f) Au-doped graphene. (g) The calculated absorption spectra of the TM-doped graphene.

Generally, the optical characteristics of a material are indeed closely related to its electronic properties. Therefore, it can be expected that the changes in those electronic properties of the TM-doped graphene will also modify their optical characteristics. Herein, the optical properties of TM-doped graphene were investigated by performing the computation of dielectric and absorption and finally the EELS spectra. The dielectric permittivity and absorption spectra for graphene and TM-doped graphene are shown in Fig. 5. For pristine graphene, both the real and imaginary parts of the dielectric permittivity are real in the low photon energy (∼0 eV) region, hindering the excitation of plasmons in the THz region. On the other hand, these TM-doped graphene exhibit negative numbers of the real parts of the dielectric permittivity, which demonstrates the possibility of plasmonic wave excitation in the THz region. From the calculated absorption spectra of TM-doped graphene in Fig. 5(g), zero absorption is observed at 0 eV due to the presence of the bandgap, although a small absorption band appeared at around 0.2–0.5 eV. This small absorption band is related to intraband transition involving electrons in the mid-gap states band δ with a relatively small number of electrons. However, two broad and intense absorption bands are observed where the first and second peaks are located at 4.3 (labeled as A) and 14.3 eV (labeled as B), respectively. The first peak originates from direct π–π* excitation, whereas the second peak is from (π + σ) excitations.^22,57–59^ Absorption is unlikely to occur in the range of 7–11 eV because of the small excitation probability for π–π*, π–σ*, or σ–π* in this photon energy range. These results are in good agreement with a similar study on alkali-metal-doped graphene.^22^ The substitution of the TM atom does not significantly change the absorption spectrum compared to that of pristine graphene, in which the B peak just slightly shifts to smaller photon energy though the bandwidth of the B peak varies depending on the TM atom.

In general, a dielectric function is a complex number given by

2 *ε*(*ω*) = *ε*_r_(*ω*) + i*ε*_i_(*ω*)

where *ε*_r_ is the real part of the dielectric function and *ε*_i_ is the imaginary part of the dielectric function. In the Quantum Espresso package, the imaginary part of the dielectric function *ε*_i_(*ω*) is calculated as a result of a response function that comes from a perturbation theory with adiabatic approximation. This imaginary part of the dielectric function *ε*_i_(*ω*) is given by:^61^3
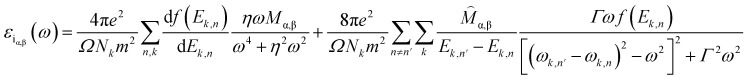
where *e* is the electron charge, *m* is the electron mass, *Ω* is the primitive cell volume, and *ω* is the incident light frequency. *E*_*k*,*n*′_ is the final state energy (empty CB) and *E*_*k*,*n*_ is the initial state (filled VB), whereas *f*(*E*_*k*,*n*′_) and *f*(*E*_*k*,*n*_)are Fermi–Dirac distribution functions for both bands. *M*_α,β_ is the moment of transition from *n* (valence states with energy *E*_*k*,*n*_) to transition *n*′ (conduction states with energy *E*_*k*,*n*′_). The real part of the dielectric function is then calculated using the well-known Kramers–Kronig transformation. The EELS spectrum is proportional to the imaginary of the inverse dielectric tensor, which is computed by using the following relationship^61^4
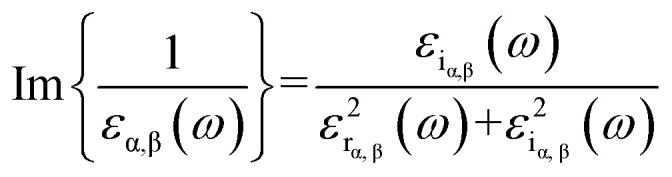


Therefore, as seen from those equations, the calculation of the EELS spectrum in this method only includes the plasmons originating from interband transitions and does not include the plasmon from intraband transitions.

**Fig. 6:**
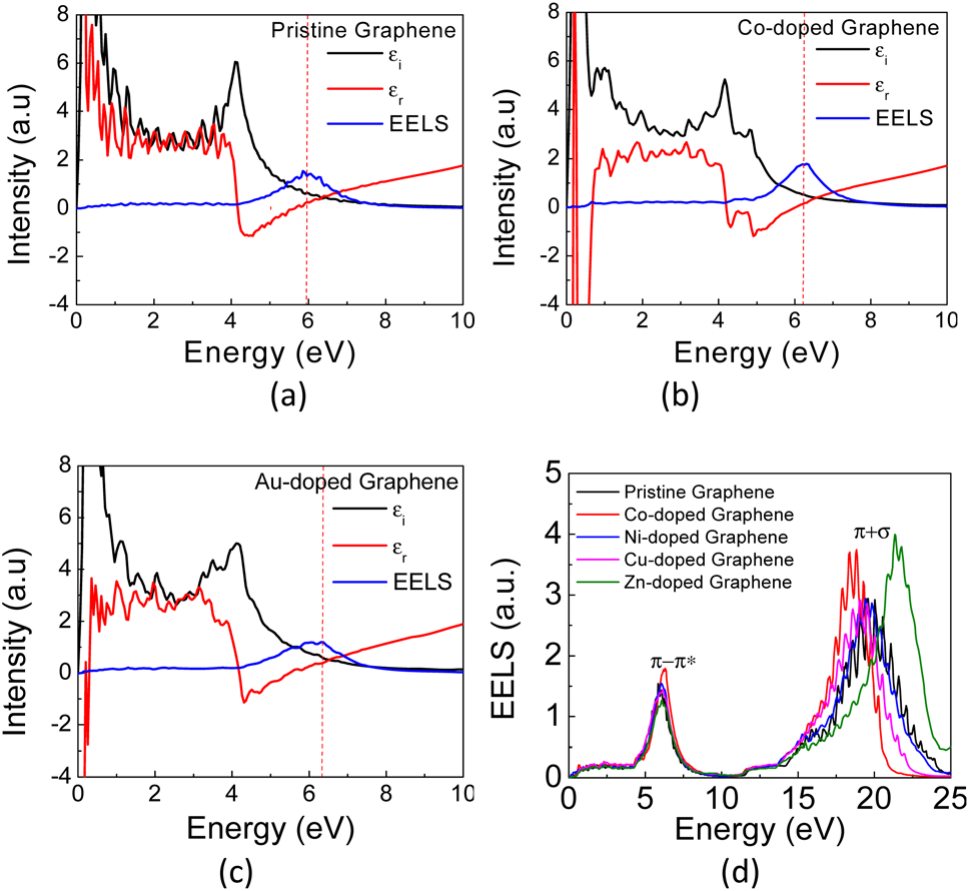
The calculated EELS spectra of (a) pristine, (b) Co- and (c) Au-doped graphene, and (d) the calculated EELS spectra of pristine graphene and TM-graphene.

In Fig. 6(a) and (b), the calculated real part of the dielectric permittivity (*ε*_1_), the imaginary part of the dielectric permittivity (*ε*_2_), and EELS spectra for Co- and Au-doped graphene are presented. As similar as seen in the absorption coefficient spectra above, the EELS spectra also exhibit two bands at different photon energies, in which the π plasmon band appears at approximately 5.9 eV, and a wider (π + σ) plasmon band appears at around 17–22 eV. This result is in good agreement with the reported values in the literature.^58^ Typically, the plasmon frequency for metallic materials can be determined considering a condition in which both the real (*ε*_1_) and imaginary parts (*ε*_2_) of the dielectric permittivity intersect the horizontal axis. Here, the EELS peak can also be used as a complementary parameter to determine the possibility of plasmon excitation, for which the EELS peak may appear at the same frequency (photon energy) as the intersection of *ε*_1_ and *ε*_2_ on the horizontal axis.^60^ However, in the case of plasmon excitation that undergoes damping due to interband transition, the plasmon frequency (photon energy) may exhibit a slight shift from the *ε*_1_ and *ε*_2_ intersection frequencies.^61^ This is the case in Fig. 6, in which the plasmon peaks in the EELS spectra do not appear exactly at the crossing point between *ε*_1_ and *ε*_2_. The substitution of TM atoms caused a slight alteration in both plasmon frequencies, as depicted in Table 2.

**Table 2:** Comparison of plasmon frequency of pristine graphene and TM-doped graphene structures obtained from EELS calculations

Graphene structure	π–π*			σ–σ*		
	*E* (eV)	*ω*_p_ (THz)	*λ*_p_ (nm)	*E* (eV)	*ω*_p_ (THz)	*λ*_p_ (nm)
Pristine	5.86	1.42	211	19.6	4.73	63.4
Co-doped	6.12	1.48	203	18.5	4.47	67.0
Ni-doped	6.09	1.47	204	19.5	4.72	63.5
Cu-doped	6.18	1.49	201	19.1	4.63	64.8
Zn-doped	6.11	1.48	203	21.3	5.25	58.2
Au-doped	6.35	1.54	195	19.7	4.76	63.0

The π plasmon frequency of pristine graphene (Fig. 6(d)) is almost unchanged due to the substitution of the TM dopant. However, the (π + σ) plasmon band differs significantly, which may indirectly indicate core-level electronic states due to hybridization with TM orbitals by involving σ bonds. This occurs in the far UV region and still has limited practical applications in the present day. On the other hand, considering the nature of the narrow band δ in these TM-doped graphene, it seems possible then to excite plasmons at low photon energies corresponding to the THz frequency range, which is associated with the intraband transition in this band.

Since the EELS calculation method cannot calculate the intraband transition, it is not seen in the calculated EELS spectra above. This intraband transition does not require large photon energy because this mid-gap states band δ behaves like a metallic band characteristic. However, as seen in DOS, only a very small number of electrons are available for this intraband transition in comparison to metals. To predict the possibility of plasmon excitation at small photon energy (∼0 eV or in the THz region) owing to this intraband transition, the following theoretical plasmon frequency was used as a rough estimation:^62^5
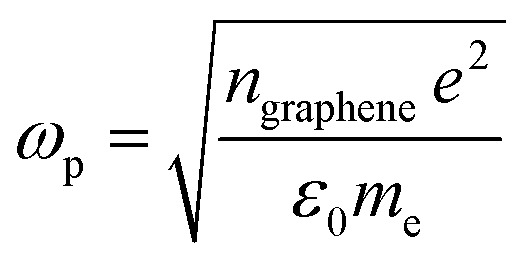
where *n*_graphene_ is the electron density inside the mid-gap states at the Fermi energy level; *e* is electron charge; *m*_e_ is electron mass, and *ε*_0_ is the vacuum permittivity. In the present stage, as a simple approximation, the electron density was obtained by integrating the mid-gap states band δ band at the Fermi energy level according to the following formula:6
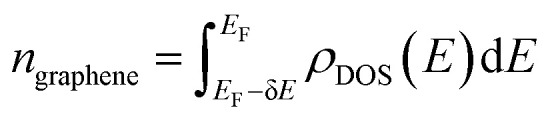
where *E*_F_ is the Fermi energy level, δ*E* is the onset bottom energy level of the mid-gap states band, where the integral boundaries are from *E*_F_ − δ*E* to *E*_F_, as indicated by two vertical dash lines in Fig. 4. From these integrations, the number of electrons per supercell for Co-, Ni-, Cu-, Zn-, and Au-doped graphene are found to be approximately 0.8, 1.5, 0.8, 1.7, and 1.5 electrons per supercell unit, respectively. The calculated plasmon frequencies *ω*_p_, using eqn (5), and the conversions in wavenumber (1/*λ*), wavelength (*λ*), and energy are listed in Table 3. These plasmon frequencies are in the range of 5–7.5 THz or equivalent to 160–250 cm^−1^ in wavenumber.

**Table 3:** Plasmons frequencies of TM-doped graphene in the terahertz region

Structure	*ω*_p_ (THz)	1/*λ* (cm^−1^)	*λ* (μm)	Energy (eV)
Co-doped graphene	5.0	168	59.3	0.021
Ni-doped graphene	7.0	232	43.0	0.029
Cu-doped graphene	5.2	172	58.0	0.021
Zn-doped graphene	7.3	245	40.8	0.030
Au-doped graphene	6.9	229	43.6	0.028

This estimated plasmon frequency range seems almost in the same range as reported by several pioneer groups who experimentally investigated and developed graphene plasmonics in the THz region.^12,65,66^ In those previous reports, graphene plasmonics are realized through Fermi energy shift by applying external electrical or magnetic fields. By adjusting the Fermi energy or the chemical potential, one can control the free electron density and electrical conductivity of graphene as well as its plasmon frequency.^67–71^ In the present work, our calculation results show that the plasmon frequency in THz range, as seen in Table 3. Here, the Fermi energy level is shifted varyingly in the mid-gap states band depending on the TM dopant.

Although the range of these plasmon frequency shifts seems small, in terms of molecular vibration energy those shifts are actually quite large. Table 3 shows the plasmon frequency is shifted in the range of 21–28 meV, equivalent to 160–250 cm^−1^. As a comparison, we may look at the following several cases. The vibration modes of water clusters in pentamer structure, involving intramolecular vibrations of H_2_O, symmetric stretching and bending as well as asymmetric stretching, appear at the THz region. The vibrational modes of water are observed at 450–780 cm^−1^, the O–H⋯O stretching vibrations are observed at 167 cm^−1^ and the O–H⋯O bending vibrations at 53 cm^−1^.^72^ Böhm *et al.* reported that in an aqueous solution the trivalent Fe cation has a different feature of THz spectra in comparison to its divalent cation. Trivalent Fe cation exhibits two absorption peaks at 180 and 260 cm^−1^, while divalent Fe cation exhibit only one absorption peak at 200 cm^−1^.^63^ The same group has also reported the same behavior for Mn and Ni.^64^ Therefore, by choosing TM-doped graphene with a plasmon frequency that matches the frequency of a specific vibration mode of a substance, a resonance between plasmon and vibration mode can be expected and possibly applied in molecular sensing or detection applications.

## 4. Conclusions

*Ab initio* calculations based on the DFT method were performed to study the structural, electronic, and optical properties of TM-doped graphene. The TM atom evaluated herein is Ni, Co, Cu, Zn, and Au, which is substituted into an 8 × 8 graphene supercell replacing one carbon atom. The structure resembles a substitutional defect by a single TM atom in the basal plane of graphene as reported elsewhere. The substitution of these TM dopants leads to a bandgap opening at the Dirac point, shifting the Fermi energy downward inside the mid-gap states band that forms the half-filled valence band top, where it varies depending on the TM atom. Such electronic-band structure changes are originated from structural/bond deformation surrounding the TM dopant along with the bond hybridization between the TM dopant and the nearest carbon atoms. In the TM-doped graphene, the absorption peaks just shift slightly toward low energies in comparison to pure graphene. The EELS calculations results confirm a plasmon frequency at approximately 5.86 eV, which is associated with the interband excitation of π electrons.

The TM-doped graphene, however, exhibit negative numbers for the real part of dielectric permittivity, which resembles metal material properties. As EELS calculations cannot predict the plasmon originating from intraband photo-excitation, by using the number of electrons in the mid-gap states band δ, the plasmon frequencies of these TM-doped graphene can be predicted to appear at the THz region around 5–7.5 THz. Although the plasmon excitation in these TM-doped graphene here is caused by a substitutional defect, which could be achieved such as by the aforementioned wet chemical process, the plasmon excitation mechanism seems principally similar to plasmonic excitation of pure graphene induced by electric or magnetic fields reported elsewhere, in which the Fermi energy shifting from Dirac cone point level leads to the possibility of intraband transition. In addition, the plasmon frequency range matches the intermolecular vibration frequencies, which is useful in THz spectroscopy for generating plasmon resonance with a particular vibration mode of a substance, the present calculation results might be beneficial for optoelectronic and molecular sensing applications in the THz region.

## Author contributions

D. U. D. investigation, formal analysis, and writing – original draft. R. H. conceptualization, formal analysis, writing – review & editing, funding acquisition. A. M. P. and K. N. methodology and writing – review & editing. All authors confirmed the final manuscript.

## Conflicts of interest

There are no conflicts to declare.

## Supplementary Material

RA-013-D2RA06446D-s001
