# Supplementary material for: Modification of plasmonic properties in several transition metal-doped graphene studied by the first principles method
Source: RSC Adv. 2023 Jan 5;13(2):1446–54. doi: 10.1039/d2ra06446d (PMC9814376; doi:10.1039/d2ra06446d)
Supplement: RA-013-D2RA06446D-s001 [file RA-013-D2RA06446D-s001.pdf]

Electronic Supplementary Material (ESI) for RSC Advances.
This journal is © The Royal Society of Chemistry 2022

Electronic Supplementary Information (ESI)

Plasmonic properties modification in several transition metal-doped graphenes studied by the first principle method

Diyan Unmu Dzujah[a], Abdul-Muizz Pradipto[a], Rahmat Hidayat[a]*, Kohji Nakamura[b]

1. In order to predict the charge and magnetic moment of transition metal in each structure, the calculations of TM-doped graphenes, as described in the main text, have been also carried out by involving the spin-polarization effect. This can be done technically by setting the *nspin* parameter in their Quantum Espresso input files to be = 2 or = 1 for the calculations with and without spin polarizations, respectively. The supercell size is 5×5 cells as shown in Figure S1, which were subjected to the optimization calculation before proceeding to further calculations. As a typical example from the calculation results with spin polarization, the PDOS for Co-doped graphene is shown in Figure S2. It is clear that the PDOS for the spin-up and the spin-down configurations are symmetric, indicating a material with approximately zero total magnetization. The charge and magnetic moment in each atom are shown in Table S1 - S6.

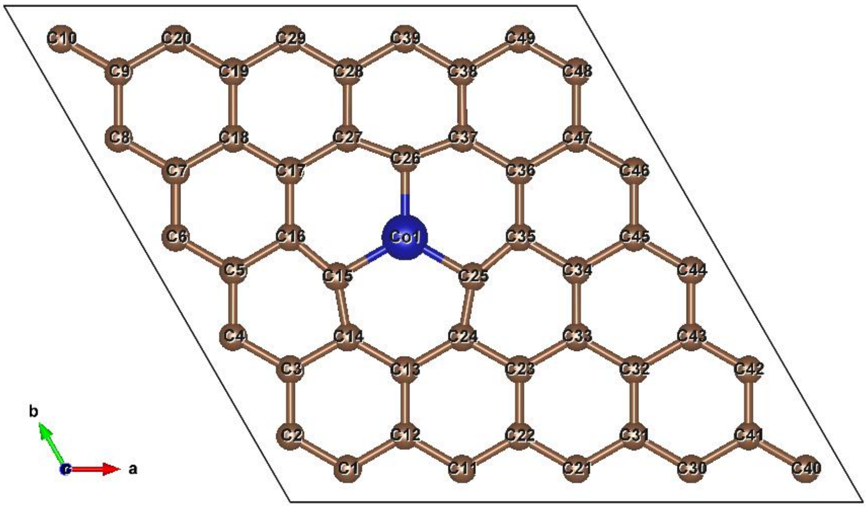

Figure S1. Structure of TM-doped graphene along with each corresponding atomic number.

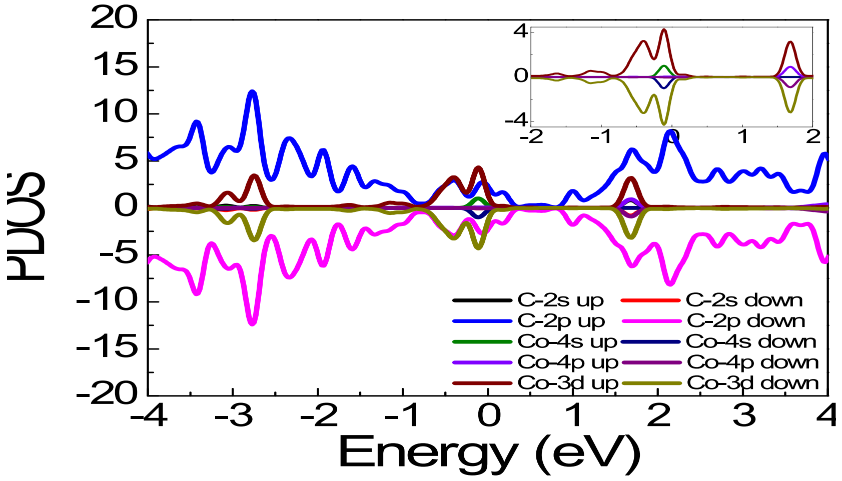

Figure S2. PDOS for Co-doped graphene calculated with spin polarization.

Table S1. Charge and magnetic moment of each atom in pristine graphene structure

Atom	Charge	Magnetic moment	Atom	Charge	Magnetic moment
C1	1.7106	-0.00010	C26	1.7106	0.00010
C2	1.7106	0.00010	C27	1.7106	0.00000
C3	1.7106	0.00000	C28	1.7106	0.00010
C4	1.7106	0.00010	C29	1.7106	0.00000
C5	1.7106	0.00000	C30	1.7106	0.00010
C6	1.7106	0.00010	C31	1.7105	0.00000
C7	1.7106	0.00000	C32	1.7105	0.00010
C8	1.7106	0.00010	C33	1.7106	0.00000
C9	1.7106	0.00000	C34	1.7105	0.00010
C10	1.7106	0.00010	C35	1.7106	0.00000
C11	1.7105	0.00000	C36	1.7105	0.00010
C12	1.7105	0.00010	C37	1.7106	0.00000
C13	1.7106	0.00000	C38	1.7106	0.00010
C14	1.7106	0.00010	C39	1.7106	0.00000
C15	1.7106	0.00000	C40	1.7106	0.00010
C16	1.7106	0.00010	C41	1.7106	-0.00010
C17	1.7106	0.00000	C42	1.7105	0.00010
C18	1.7106	0.00010	C43	1.7106	0.00000
C19	1.7106	0.00000	C44	1.7106	0.00010
C20	1.7106	0.00010	C45	1.7106	0.00000
C21	1.7105	0.00000	C46	1.7105	0.00010
C22	1.7105	0.00010	C47	1.7106	0.00000
C23	1.7106	0.00000	C48	1.7106	0.00010
C24	1.7106	0.00010	C49	1.7106	-0.00010

C25	1.7106	0.00000	C50	1.7106	0.00010

Valence electron of Carbon = $2s^2\ 2p^2$

Total Magnetization = 0.00 Bohr mag/cell

Table S2. Charge and magnetic moment of each atom in the Co-doped graphene structure

Atom	Charge	Magnetic moment	Atom	Charge	Magnetic moment
C1	1.4311	0.0000	Co	5.8982	-0.0002
C2	1.43	0.0000	C26	1.5152	-0.0001
C3	1.4308	0.0000	C27	1.4772	0.0000
C4	1.4354	0.0000	C28	1.4309	0.0000
C5	1.4309	0.0000	C29	1.43	0.0000
C6	1.43	0.0000	C30	1.43	0.0000
C7	1.4312	0.0000	C31	1.4322	0.0000
C8	1.4304	0.0000	C32	1.4301	0.0000
C9	1.4327	0.0000	C33	1.4354	0.0000
C10	1.4304	0.0000	C34	1.4309	0.0000
C11	1.4312	0.0000	C35	1.4773	0.0000
C12	1.4303	0.0000	C36	1.4369	0.0000
C13	1.437	0.0000	C37	1.4772	0.0000
C14	1.4773	0.0000	C38	1.4309	0.0000
C15	1.5148	-0.0001	C39	1.4354	0.0000
C16	1.4772	0.0000	C40	1.4295	0.0000
C17	1.4368	0.0000	C41	1.4322	0.0000
C18	1.4304	0.0000	C42	1.4301	0.0000
C19	1.431	0.0000	C43	1.4322	0.0000
C20	1.4304	0.0000	C44	1.4295	0.0000
C21	1.4295	0.0000	C45	1.43	0.0000
C22	1.43	0.0000	C46	1.4312	0.0000
C23	1.4308	0.0000	C47	1.4304	0.0000
C24	1.4773	0.0000	C48	1.431	0.0000
C25	1.5149	-0.0001	C49	1.43	0.0000

The valence electrons of Co = $4s^2\ 3d^7$ (=9*e*), the valence charge on the Co site = 5.8982*e*.

Total Magnetization: 0.00 Bohr mag/cell

Table S3. Charge and magnetic moment of each atom in the Ni-doped graphene structure

Atom	Charge	Magnetic moment	Atom	Charge	Magnetic moment
C1	1.592	0.0001	Ni	6.9484	-0.0012
C2	1.6007	-0.0001	C26	1.6108	0.0004
C3	1.5957	0.0002	C27	1.6026	-0.0002
C4	1.5918	-0.0001	C28	1.5957	0.0002
C5	1.5956	0.0002	C29	1.6007	-0.0001
C6	1.6006	-0.0001	C30	1.5993	0.0001
C7	1.592	0.0001	C31	1.5956	-0.0001
C8	1.5956	-0.0001	C32	1.5992	0.0001
C9	1.598	0.0000	C33	1.5918	-0.0001
C10	1.5956	-0.0001	C34	1.5957	0.0002
C11	1.592	0.0001	C35	1.6027	-0.0002
C12	1.5916	-0.0001	C36	1.5853	0.0001
C13	1.5852	0.0001	C37	1.6027	-0.0002
C14	1.6025	-0.0002	C38	1.5958	0.0002
C15	1.6105	0.0004	C39	1.5918	-0.0001
C16	1.6026	-0.0002	C40	1.6019	0.0001
C17	1.5852	0.0001	C41	1.5956	-0.0001
C18	1.5917	-0.0001	C42	1.5992	0.0001
C19	1.5921	0.0001	C43	1.5956	-0.0001
C20	1.5956	-0.0001	C44	1.6018	0.0001
C21	1.6018	0.0001	C45	1.6006	-0.0001
C22	1.6006	-0.0001	C46	1.5921	0.0001
C23	1.5957	0.0002	C47	1.5917	-0.0001
C24	1.6025	-0.0002	C48	1.5921	0.0001
C25	1.6106	0.0004	C49	1.6007	-0.0001

The valence electrons of Ni = $4s^2 3d^8$ (=10*e*), the total charge on the Ni site = 6.9484*e*.

Total Magnetization: 0.00 Bohr mag/cell

Table S4. Charge and magnetic moment of each atom in the Cu-doped graphene structure

Atom	Charge	Magnetic moment	Atom	Charge	Magnetic moment
C1	1.467	0.0017	Cu	7.8941	0
C2	1.4706	-9.00E-04	C26	1.527	0.0069
C3	1.4625	0.0014	C27	1.5206	-0.0014
C4	1.464	-5.00E-04	C28	1.502	0.0016

C5	1.4624	0.0014	C29	1.5005	-1.00E-03
C6	1.4706	-9.00E-04	C30	1.48	0.0022
C7	1.467	0.0016	C31	1.4757	-1.00E-03
C8	1.4661	-0.0014	C32	1.48	0.0022
C9	1.4662	0.0073	C33	1.4729	-8.00E-04
C10	1.4661	-0.0014	C34	1.4739	0.0012
C11	1.4676	0.0038	C35	1.455	-0.0016
C12	1.4722	-0.0012	C36	1.4228	0.0103
C13	1.458	0.0066	C37	1.4549	-0.0016
C14	1.4575	-0.0012	C38	1.4738	0.0012
C15	1.4328	0.0052	C39	1.4729	-8.00E-04
C16	1.4575	-0.0012	C40	1.4729	0.002
C17	1.4581	0.0066	C41	1.4724	-8.00E-04
C18	1.4723	-0.0012	C42	1.472	8.00E-04
C19	1.4676	0.0038	C43	1.4725	-8.00E-04
C20	1.4744	-0.0015	C44	1.4729	0.002
C21	1.4959	0.002	C45	1.4742	-0.0011
C22	1.5005	-1.00E-03	C46	1.4586	0.0042
C23	1.502	0.0016	C47	1.4499	-0.0018
C24	1.5205	-0.0014	C48	1.4585	0.0042
C25	1.527	0.0069	C49	1.4741	-0.0011

Valence electron of Cu = $4s^2\,3d^9$; the total charge on the Cu site = 7.8941*e*.

Total Magnetization: 0.20 Bohr mag/cell

Table S5. Charge and magnetic moment of each atom in Zn-doped graphene structure

Atom	Charge	Magnetic moment	Atom	Charge	Magnetic moment
C1	1.5175	0.0030	Zn	8.901	0.1089
C2	1.5342	0.0013	C26	1.5773	0.0515
C3	1.5272	0.0179	C27	1.5519	-0.0037
C4	1.5181	-0.0035	C28	1.549	0.0056
C5	1.5272	0.0179	C29	1.5478	-0.0020
C6	1.5342	0.0013	C30	1.5338	0.0031
C7	1.5175	0.0030	C31	1.5276	-0.0023
C8	1.5201	-0.0025	C32	1.5339	0.0031
C9	1.5187	0.0096	C33	1.5231	-0.0022
C10	1.5201	-0.0025	C34	1.5344	0.0089
C11	1.518	0.0057	C35	1.5176	0.0077
C12	1.5203	-0.0029	C36	1.4811	0.0190
C13	1.4998	0.0166	C37	1.5177	0.0076
C14	1.5228	0.0026	C38	1.5344	0.0089

C15	1.537	0.1758	C39	1.5231	-0.0022
C16	1.5225	0.0026	C40	1.5333	0.0048
C17	1.4998	0.0166	C41	1.5259	-0.0022
C18	1.5203	-0.0029	C42	1.5295	0.0017
C19	1.5179	0.0057	C43	1.5259	-0.0022
C20	1.5245	-0.0026	C44	1.5333	0.0048
C21	1.5452	0.0032	C45	1.5349	0.0003
C22	1.5478	-0.0020	C46	1.5128	0.0058
C23	1.549	0.0056	C47	1.5094	-0.0031
C24	1.552	-0.0037	C48	1.5129	0.0059
C25	1.5772	0.0520	C49	1.5349	0.0003

Valence electron of Zn = $4s^2$ $3d^{10}$, the total charge on the Zn site = 8.901*e*.

Total Magnetization: 1.25 Bohr mag/cell

Table S6. Charge and magnetic moment of each atom in Au-doped graphene structure

Atom	Charge	Magnetic moment	Atom	Charge	Magnetic moment
C1	1.5392	0.0000	Au	5.6369	0.1116
C2	1.5609	0.0021	C26	1.6037	0.1618
C3	1.5563	0.0062	C27	1.5596	0.0033
C4	1.5371	-0.0006	C28	1.5601	0.0184
C5	1.5424	0.0003	C29	1.5623	0.0039
C6	1.5472	-0.0006	C30	1.5556	0.0003
C7	1.5347	0.0004	C31	1.5444	-0.0008
C8	1.5393	-0.0001	C32	1.5509	0.0002
C9	1.5437	-0.0002	C33	1.537	-0.0006
C10	1.5433	-0.0003	C34	1.5424	0.0002
C11	1.5392	0.0000	C35	1.5325	-0.0013
C12	1.5432	-0.0002	C36	1.5096	0.0095
C13	1.5298	0.0033	C37	1.5598	0.0034
C14	1.5661	0.0094	C38	1.5602	0.0184
C15	1.5613	0.0337	C39	1.5404	-0.0018
C16	1.5324	-0.0013	C40	1.561	0.0023
C17	1.5095	0.0095	C41	1.5444	-0.0008
C18	1.531	-0.0003	C42	1.5509	0.0002
C19	1.5347	0.0002	C43	1.5427	-0.0007
C20	1.5433	-0.0003	C44	1.549	0.0001
C21	1.561	0.0023	C45	1.5472	-0.0006
C22	1.5608	0.0020	C46	1.5347	0.0005
C23	1.5562	0.0061	C47	1.5311	-0.0003
C24	1.5659	0.0094	C48	1.5347	0.0002

C25	1.5611	0.0330	C49	1.5624	0.0039

The valence electrons of Au = $6s^1 5d^{10}$ (=11*e*), the valence charge on the Au site = 5.6369*e*.

Total Magnetization: 1.00 Bohr mag/cell

Based on the above calculation data, all transition metal dopants, except Au, losses almost 3*e* which looks like a cation with around 3+ charge. Those 3*e* are transferred and involved in the formation of hybridization with the three nearest carbon atoms in the graphene structure. Due to the nature of graphene with a high electron delocalization system, the transferred electrons are also delocalized. Since the magnetic moment for all atoms is very small, the total magnetizations of these TM-doped graphenes are almost zero, leading to a non-magnetic material.

2. While their total magnetizations are almost zero, it is still important to verify whether the spin polarization effect can cause different characteristics on the calculation results of dielectric and EELS spectra. For that purpose, the spectra for Co-doped graphene have been calculated with and without involving spin polarization. As seen in Figure S3, the dielectric and EELS spectra calculated with and without spin polarization have almost similar shapes and characteristics, such as the EELS peaks that appear around 6.166 eV (1,491 THz) and 6.121 eV (1,480 THz), respectively. In addition, their absorption spectra show also similar spectral shapes peaked at A = 4.800 eV, B = 13.791 eV and A = 4.850 eV, B = 13.732 eV for calculation results with spin-polarization and without spin-polarization, respectively. Moreover, the total energy for the Co-doped graphene structure with spin-polarization is -2640.28927220 Ry and without spin-polarization is -2640.28940001 Ry. Therefore, the calculated optical characteristics of these TM-doped graphenes are not significantly affected by spin polarization.

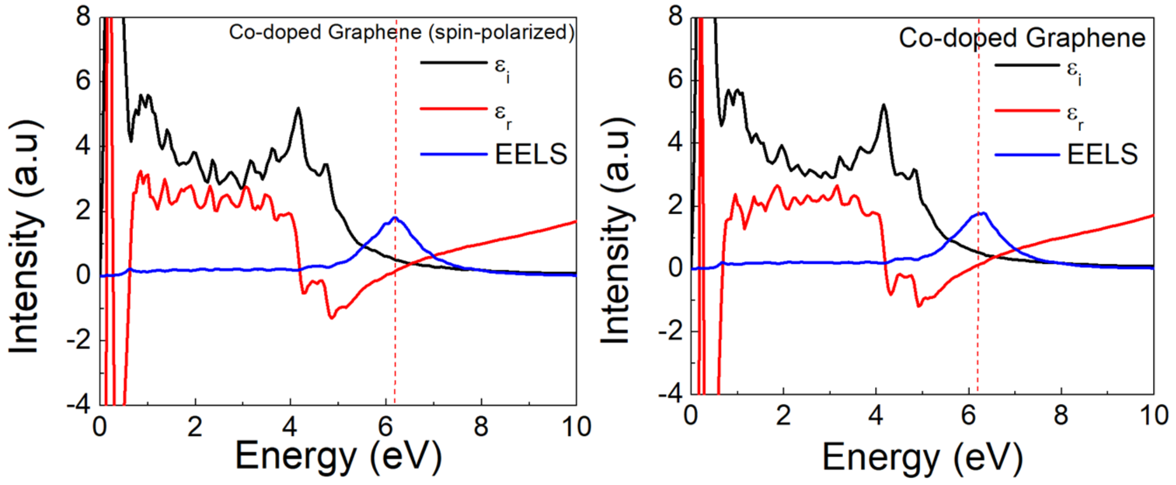

(a) (b)

Figure S3. The calculated dielectric constants and EELS spectra of Co-doped graphene (a) with spin-polarization and (b) without spin-polarization.

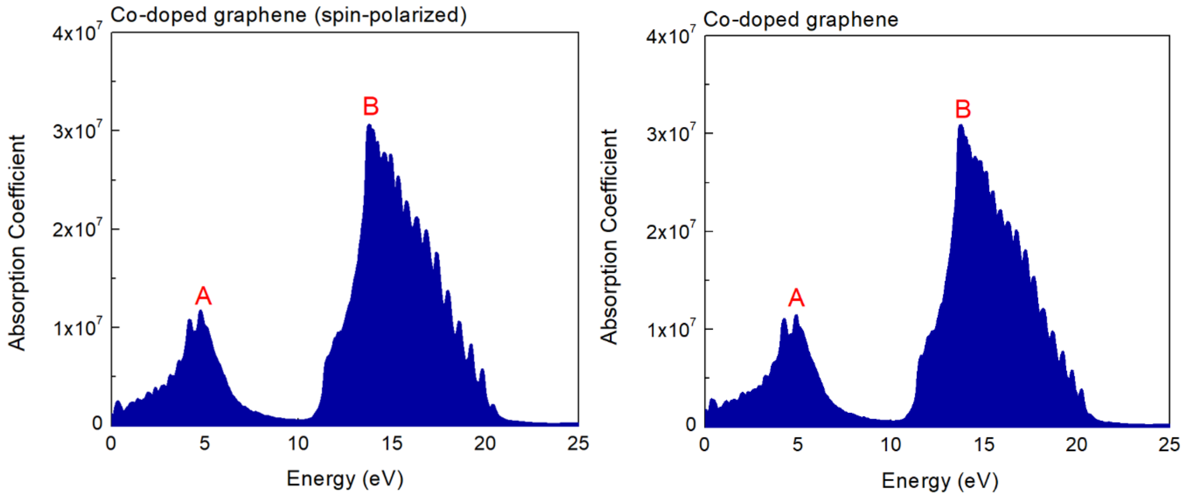

(a) (b)

Figure S4. The calculated absorption coefficient of Co-doped graphene (a) with spin-polarization and (b) without spin-polarization effect.
